# Quantitative evaluation of pediatric umbilical loop stomas: 2 decades of experience from a single institution

**DOI:** 10.1007/s00383-023-05546-3

**Published:** 2023-09-07

**Authors:** Daisuke Ishii, Yuka Kumata, Seiya Ishii, Keita Motoki, Hisayuki Miyagi

**Affiliations:** Division of Pediatric Surgery, Department of Surgery, Asahikawa Medical University, 2-1-1, Midorigaoka-Higashi, Asahikawa, 078-8510 Japan

**Keywords:** Umbilical stoma, Umbilical colostomy, Umbilical ileostomy, Stoma, Postoperative scar

## Abstract

**Purpose:**

Since pediatric stomas are often temporary, their creation, management, and closure should be simple, with minimal complications and excellent cosmetic results. We began employing umbilical stomas in 2000. This study aimed to characterize the ingenuity and utility of umbilical stomas and provide a quantitative evaluation of their cosmetic outcomes.

**Methods:**

We examined cases of stoma construction and closure surgery performed in our department from January 2000 to December 2022. The umbilical and non-umbilical stoma groups included 54 and 42 cases, respectively, and the findings for both groups were compared and analyzed.

**Results:**

The two groups showed no significant differences in the incidence of complications. The Manchester Scar Scale score for the umbilical stoma group (8.42 ± 1.85) was significantly better than that for the non-umbilical stoma group (16.31 ± 2.96; *P* < 0.01). Likewise, in Patient and Observer Scar Assessment Scale assessments, the umbilical stoma group showed significantly better scores in both the observer scale (9.48 ± 2.50 vs. 21.78 ± 7.26; *P* < 0.01) and the patient scale (10.5 ± 1.39 vs. 22.40 ± 7.35; *P* < 0.01).

**Conclusions:**

Umbilical stomas are easy to manage and yield an inconspicuous closure incision with excellent cosmetic outcomes. Although patient selection is important, pediatric umbilical stomas are a valuable option that can be actively employed.

## Introduction

Stoma construction is essential and life-saving not only for patients with rectoanal anomalies and Hirschsprung’s disease, but also for children with intestinal perforation, necrosis, and obstruction [1, 2]. Stomas constructed during childhood are often temporary, highlighting the importance of easy construction, management, and closure with minimal complications and excellent cosmetic results. With the declining mortality rate because of advancements in treatments for various pediatric surgical diseases, even in neonates, reducing the size and visibility of surgical wounds has become more important to improve the quality of life (QOL) of patients during their growth and development [3, 4].

Umbilical stoma construction was first reported in 1980 by Turnbull et al., who performed a permanent colostomy at the umbilicus during a Miles operation (abdominoperineal resection) for rectal cancer [5]. In the field of pediatric surgery, umbilical stoma construction for Hirschsprung’s disease was reported by Cameron et al. in 1982, and the effectiveness of this approach was reported by Fitzgerald et al., from the same group, in 1989, based on their experience with 47 cases [6, 7].

We began implementing umbilical stoma construction at our institution in 2000 and have achieved good functional and cosmetic results. However, the literature on umbilical stomas is quite limited [8–10], and very few reports have described quantitative evaluations of their cosmetic outcomes. Therefore, this study aimed to examine the ingenuity and usefulness of umbilical stomas and to report on their cosmetic outcomes quantitatively.

## Materials and methods

### Patient selection

We studied cases involving stoma construction and closure at our institution between January 2000 and December 2022. We compared the findings from 54 cases of umbilical stoma construction with those of 42 cases of non-umbilical stoma construction.

### Quantified variables

For both groups, we examined the causes of stoma construction, the intestinal segment used for construction, patient age and weight at the times of construction and closure, operation time, blood loss, duration of stoma construction, follow-up period, complications, and the Manchester Scar Scale (MSS) and Patient and Observer Scar Assessment Scale (POSAS) scores, based on medical records and interviews.

The MSS was developed by Beausang et al. [11] in 1998. This scale evaluates scar color, surface appearance (matte vs. shiny), contour, distortion, and texture, with larger values indicating greater scar severity. The MSS is a sensitive method of scar evaluation and is considered effective for quantifying the severity of various scars. The POSAS is a questionnaire that was developed to assess scar quality. It is a partially observer-administered (Observer Scale) and partially patient self-administered scale (Patient Scale) and includes scar characteristics that are considered clinically important. The observer score includes six items: vascularization, pigmentation, thickness, surface roughness, pliability, and surface area. Independently, the patient is asked to score pain, pruritus, color, thickness, relief, and pliability [12, 13]. The POSAS is an innovative scale that emphasizes the patient’s opinion, which has been reported to be particularly influenced by itching and scar thickness. The POSAS is considered an appropriate and reliable tool for complete scar evaluation. Nowadays, the scale has been adopted throughout the world in various fields of surgery and dermatology [14]. Both the MSS and POSAS are commonly used for quantitative evaluation of cosmetic outcomes in pediatric patients and for assessment of surgical scars [15–17].

### Protocol for umbilical stoma construction

Patients with a poor general condition, low-positioned umbilicus, umbilical infection, and multiple stomas were considered unsuitable for umbilical stoma construction.

**Fig.1:**
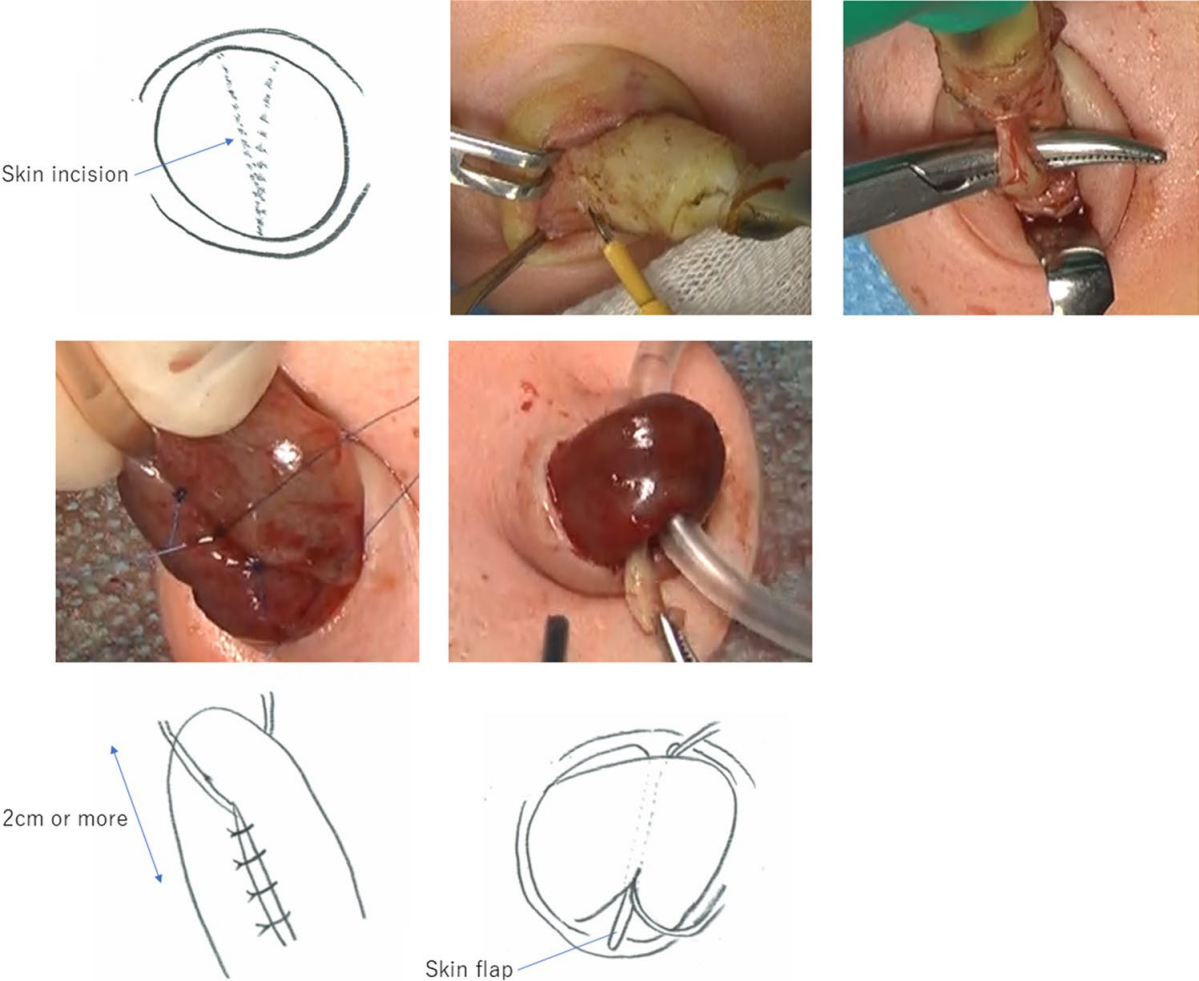
Umbilical stoma construction. (1-1) For cases showing post-umbilical detachment, a “V-shaped” skin incision was made in the umbilical area. (1-2) In cases with pre-umbilical detachment, the periumbilical skin was maximally preserved and the umbilicus was hollowed out at the umbilical transition. (1-3) The umbilical arteries and veins, and the remnants of the urachus, were ligated and divided at a sufficient distance. (1-4) The distal and proximal sides of the intestine were sutured to the front and back of the mesentery with four non-absorbable monofilament sutures each to prevent stomal prolapse. (1-5) The “V-shaped” skin was trimmed, inserted, and fixed between the stomal limbs as a skin flap, preventing stomal prolapse and recession

**Fig.2:**
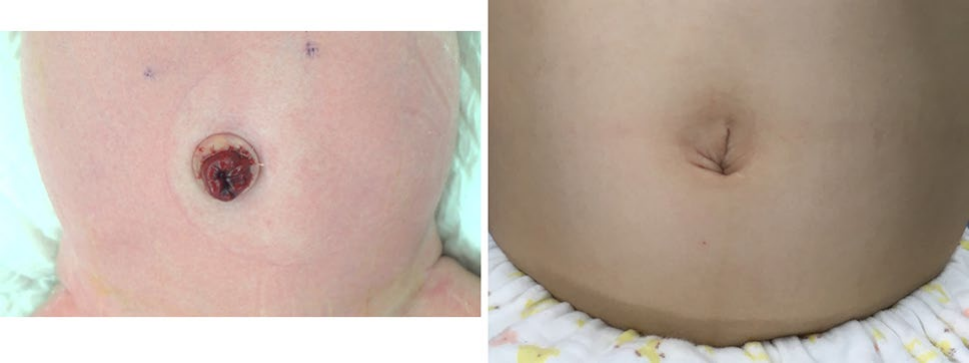
Umbilical stoma and cosmetic outcome after closure. (2-1) An umbilical transverse loop colostomy was created on day 0 for an intermediate anorectal malformation with a rectobulbar fistula. (2-2) The patient underwent colostomy closure at the age of 8 months after radical surgery

For cases showing post-umbilical detachment, a “V-shaped” skin incision was made in the umbilical area (Fig. 1) following the longitudinal or transverse lines, and the linea alba was incised longitudinally. In cases with pre-umbilical detachment, the periumbilical skin was maximally preserved, and the umbilicus was hollowed out at the umbilical transition (Fig. 1). Next, the umbilical arteries and veins, and the remnants of the urachus, were ligated and divided at a sufficient distance (Fig. 1), and the intestine for stoma construction was brought out through the abdomen. To prevent stomal prolapse, the distal and proximal sides of the intestine were sutured to the front and back of the mesentery with four non-absorbable monofilament sutures each (Fig. 1). In addition, the intestine and fascia were fixed with four or more non-absorbable monofilament sutures. When suturing the intestine and fascia, the distance between the fascial fixation point and the stoma opening was maintained at ≥ 2 cm to ensure adequate stoma height. Subsequently, the stoma was created using Turnbull’s modified method [5] (three-point fixation of the intestinal end, intestinal wall, and skin; formation of skin flaps; and primary opening). During this process, the “V-shaped” skin was trimmed, inserted, and fixed between the stomal limbs as a skin flap (Fig. 1), preventing stomal prolapse and recession. Our umbilical stoma is shown in Fig. 2.

### Umbilical stoma closure

When performing umbilical reconstruction during the closure of the umbilical stoma, adequate thickness of the surrounding adipose tissue and the availability of excess skin was confirmed. The closure procedure began with a skin incision around the stoma, which was performed while keeping skin excision to a minimum. The adhesion between the subcutaneous tissue near the intestine and the abdominal wall was carefully separated, preserving as much of the subcutaneous tissue as possible. The stoma section of the intestine was sufficiently separated up to the wound edge, and intestinal resection was kept to a minimum. Anastomosis was performed using a hand-sewn anastomosis with Albert–Lembert two-layer end-to-end suturing as the foundation.

The peritoneum and fascia were closed using absorbable interrupted sutures. For skin suturing, the wound was thoroughly cleaned under aseptic conditions, and the dermis and fascia were sutured and fixed with monofilament absorbable sutures to create a deep and natural umbilical fossa (Fig. 2). The wound was then compressed with a cotton ball for umbilical reconstruction purposes and closed with a dressing film for several days, taking care to prevent infection.

### Non-umbilical stoma

Stoma placement was determined by the pathophysiology and the principles of the Cleveland Clinic. The skin incisions were transverse in many cases. In all cases, a stoma was not created in the laparotomy wound. The creation of the non-umbilical stoma used the same method as that of the umbilical stoma, but a skin flap was not used. At the time of non-umbilical stoma closure, the skin incision should be annular, and skin suturing during stoma closure used purse-string skin sutures in many cases.

### Statistical analyses

Statistical analyses were performed using EZR (Easy R, Ver. 1.41) [18]. This software, which is based on R and R commander, is freely available at http://www.jichi.ac.jp/saitama-sct/SaitamaHP.files/statmed.html and runs on Windows (Microsoft Corporation, USA). Fisher's exact test and the *t*-test were used for univariate analyses. Statistical significance was set at *P* < 0.05.

## Results

The results are presented in Table 1. We compared the findings from 54 cases with umbilical stomas and 42 with non-umbilical stomas (right upper abdomen, 28 cases; left upper abdomen, 14 cases). The most common reason for stoma construction in the umbilical stoma group was anorectal malformations (ARMs), which were significantly more frequent in this group than in the non-umbilical stoma group (37 vs. 11 cases, *P* < 0.01). In the non-umbilical stoma group, intestinal perforation was the most common reason and was significantly more frequent than in the umbilical stoma group (1 vs. 19 cases, *P* < 0.01). The transverse colon was the most constructed intestinal segment in the umbilical stoma group, and transverse colon construction was significantly more frequent in the umbilical stoma group than in the non-umbilical stoma group (41 vs. 19 cases, *P* < 0.01). In contrast, the small intestine was the most constructed intestinal segment in the non-umbilical stoma group, and small intestine construction was significantly more frequent in the non-umbilical stoma group than in the umbilical stoma group (7 vs. 22 cases, *P* < 0.01).

**Table 1:** Results

		Umbilical stoma	Non-umbilical stoma	*P* value
*N*		54	42 Right upper quadrant (28) Left upper quadrant (14)	0.77
Male/Female		25 / 29	17/25	0.25
Primary disease	Anorectal malformation Hirschsprung’s disease intestinal atresia intestinal perforation intestinal obstruction	37 15 1 1 0	11 8 2 19 2	< 0.01 0.61 0.59 < 0.01 0.20
Intestinal segment	Ileum transverse colon sigmoid colon	7 42 5	22 19 1	< 0.01 < 0.01 0.21
Construction Age Body weight Operative time Volume of bleeding	(Months) (kg) (min) (g)	1.58 ± 3.15 3.69 ± 1.45 60.24 ± 20.12 1.42 ± 3.32	1.79 ± 8.52 2.14 ± 1.95 65.81 ± 21.74 7.97 ± 10.39	0.85 < 0.01 0.28 < 0.01
Closure Age Body weight Operative time Volume of bleeding	(Months) (kg) (min) (g)	7.00 ± 1.01 7.17 ± 2.13 91.09 ± 20.12 6.69 ± 7.44	5.54 ± 3.32 9.63 ± 9.66 98.67 ± 24.90 9.01 ± 11.63	< 0.05 0.12 0.45 0.25
Complication Stomal prolapse Parastomal hernia Stomal recession Stomal falling		7 3 2 0	5 1 1 2	0.85 0.16 0.35 0.21
During of stoma	(Days)	174.27 ± 89.97	245.49 ± 275.76	0.11
Follow up period	(Years)	9.18 ± 6.37	10.26 ± 5.78	0.29
Cosmetic outcome Manchester scar scale Patient and observer Scar–assessment scale	Observer scale Patient scale	8.24 ± 1.85 9.48 ± 2.50 10.25 ± 1.39	16.31 ± 2.96 21.78 ± 7.26 22.40 ± 7.35	< 0.01 < 0.01 < 0.01

In a comparison of the umbilical and non-umbilical enterostomy groups, the mean age at construction was 1.58 ± 3.15 vs. 1.79 ± 8.52 months (*P* = 0.85), mean body weight at construction was 3.69 ± 1.45 vs. 2.14 ± 1.95 kg (*P* < 0.01), mean operative time for construction was 60.24 ± 20.54 vs. 65.81 ± 21.74 min (*P* = 0.28), mean age at closure was 7.00 ± 1.01 vs. 5.54 ± 3.32 months (*P* < 0.05), mean operative time for closure was 91.09 ± 20.12 vs. 98.67 ± 24.09 min (*P* = 0.45), and duration of enterostomy was 174.27 ± 89.97 vs. 245.49 ± 275.76 days (*P* = 0.11). Complications in the umbilical stoma group included stomal prolapse in seven cases (13.0%), parastomal hernia in three cases (5.6%), and stomal recession in two cases (3.7%). None of the cases showed stomal falling. In the non-umbilical stoma group, two cases (4.8%) required reconstruction due to stomal falling. The two groups showed no significant differences in the incidence of any of the complications.

The MSS score in the umbilical stoma group was 8.42 ± 1.85, which was significantly better than that in the non-umbilical stoma group (16.31 ± 2.96; *P* < 0.01). In assessments performed using the POSAS, both the observer scale score (9.48 ± 2.50 vs. 21.78 ± 7.26; *P* < 0.01) and the patient scale score (10.5 ± 1.39 vs. 22.40 ± 7.35; *P* < 0.01) were significantly better in the umbilical stoma group.

## Discussion

In this study, we quantitatively demonstrated that umbilical stoma closure was significantly more cosmetic than non-umbilical stoma wound closure, although the indications were selective. In addition, the two groups showed no significant differences in the incidence of any of the complications.

The usefulness and cosmetic acceptability of umbilical stoma have been reported in other studies, with similar results. Cameron et al. [6] and Fitzgerald et al. [7] reported on the use of divided umbilical colostomies in nine cases of high ARMs with rectourethral fistulae. The proximal stoma was brought out at the umbilicus, with the distal mucous fistula in the left lower abdomen. These authors were thus the first to create colostomies at the umbilicus in patients with ARMs. After colostomy closure, the resulting scar closely resembled a normal umbilicus, and was cosmetically superior to the scar of a colostomy placed elsewhere. In 2012, Hamada et al. [9] first reported on a temporary umbilical loop colostomy procedure for intermediate ARMs. The loop was divided 7 days postoperatively to stop fecal flow toward the distal rectal pouch and prevent fecal impaction. The colostomy was closed 2–3 months after posterior sagittal anorectoplasty through the peristomal skin incision, followed by end-to-end anastomosis. Healing of umbilical wounds after stoma closure was excellent.

In children, the area of the abdominal wall is smaller, making the size of stoma appliances relatively larger and causing restrictions on the attachment site and area, which can lead to skin problems and make management difficult [19]. Children may experience pouch problems due to developmental rib arch and lower abdominal creases, but the umbilicus is well away from these areas and has a large surface area for easy management of these problems. Additionally, for an umbilical stoma, the navel is located at the center of the abdomen, allowing for sufficient fixation between the stoma appliance and the skin even in small children, resulting in fewer skin problems and easier management [6, 7, 20].

Another advantage of the umbilical stoma is that when it is closed, the scar becomes inconspicuous as it merges with the navel’s indentation and wrinkles, leaving no surgical scars related to the stoma on the abdomen [9]. In this study, the MSS and POSAS were used to evaluate the cosmetic outcomes. The cosmetic outcomes of surgical scars and QOL are often related in adults [21–23]. Brown reported that the clinician's objective scar rating differed significantly from the patient-rated scar severity in adult patients (> 16 years) with heterogenous types of scars presenting at an outpatient department, and that the latter rating correlated with subjective psychological distress [24]. In children, reducing the size and visibility of surgical scars can improve QOL during growth and development [3, 4]. This report demonstrated that umbilical stomas yielded excellent cosmetic outcomes, suggesting that these stomas may also be beneficial for the QOL of affected children.

In this study, the exclusion criteria for umbilical stoma creation included poor general condition, low navel position, navel infection, and the presence of duplicate stomas. As a result, non-umbilical stomas were chosen for many cases with prematurity, low birth weight, and intestinal perforation, which may explain the significant intergroup differences in terms of reasons for stoma creation, weight at creation, and age at closure. In the non-umbilical stoma group, the stoma was created at a lower body weight, which may have resulted in a relatively larger stoma closure wound. The different timing of closure may have affected wound healing. In the non-umbilical stoma group, the wound edges may be difficult to fit, because we skin suturing during stoma closure used purse-string skin sutures in many cases. But purse-string skin sutures haves been reported to have less surgical site infections [25], shorter wound length [26], and better cosmetic results [27] compared with straight closures, so we chose that method. The flat abdominal wall or the depressed umbilical region may have had the greatest influence on the results, but the possibility of bias, as described above, remains an issue for further investigation, including the expansion of this technique.

Umbilical and non-umbilical stomas showed no significant differences in the incidence of complications, and none of the cases in the umbilical stoma group in this study needed reconstruction. Regarding the complications of umbilical stomas, Thorlakson et al. reported on two cases of peristomal hernia and 3 cases of stoma prolapse among 150 adult cases [28], while Cameron et al. [6] and Fitzgerald et al. [7] reported no specific complications in pediatric cases. Hamada et al. reported natural sinking and stenosis of the stoma due to natural closure of the umbilical ring [9]. We have no experience with stoma stenosis due to spontaneous closure of the umbilical ring in umbilical colostomies. The size of the fascia hole depends on the patient’s physique, but it should be large enough to allow insertion of the little finger, and if it is narrow, a fasciotomy is performed. The stoma site is marked in accordance with the five principles established by the Cleveland Clinic: (1) a position lower than the navel, (2) a position that penetrates the rectus abdominis muscle, (3) a position at the apex of the abdominal fat layer, (4) a position that avoids skin depressions, wrinkles, scars, and proximity to the anterior superior iliac spine, and (5) a position where the individual can see and easily perform self-care [29]. In umbilical stomas that do not follow these principles, the main issues are related to the lack of penetration of the rectus abdominis muscle, stomal prolapse due to the concentration of abdominal pressure in the navel area, and retraction of a stoma that is not at the apex of the abdominal fat layer. As a preventive measure against stomal prolapse (or stomal falling), the peripheral and central sides of the intestine are sutured and fixed with four stitches each of non-absorbable monofilament thread on both sides of the mesentery, and the intestine and fascia are fixed with four or more stitches of non-absorbable monofilament thread. In addition, trimmed skin is inserted between the stomal limbs as a skin flap. As a preventive measure against stoma retraction, the fascial fixation and stoma opening are set at least 2 cm apart to ensure sufficient stoma height.

**Fig.3:**
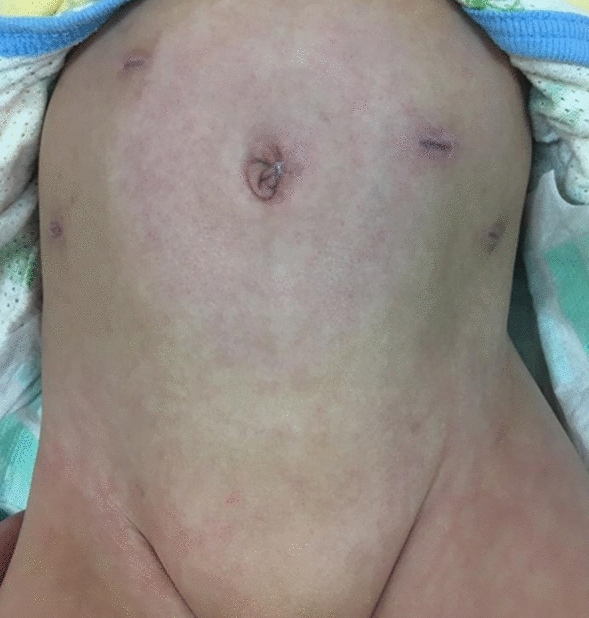
Case of a three-stage LAARP using an umbilical stoma for cases with a high imperforate anus

Many surgeries utilizing the navel have been reported recently. The umbilical incision was initially developed for hypertrophic pyloric stenosis surgery but has since been widely used for ileal and jejunal atresia, duodenal atresia, Meckel’s diverticulum, ovarian cysts, and intussusception in newborns [30]. Laparoscopic-assisted anorectal pull-through for ARMs [31] and laparoscopic surgery for Hirschsprung’s disease [32, 33] are now commonly performed with the first port placed in the umbilical region. In this respect, cases with an intermediate imperforate anus that do not require intra-abdominal manipulation with sacroperineal procedures, lone and diverting stomas are considered the most effective indications for umbilical stomas [9, 10]. However, we have previously described a three-stage laparoscopic assisted anorectoplasty (LAARP) using an umbilical stoma for cases with a high imperforate anus, demonstrating its minimally invasive and excellent cosmetic properties (Fig. 3) [34]. In this report, the first port was placed in the left upper abdomen using Hasson’s open-entry technique, and the surgery was performed safely. Xu et al. reported on a two-stage laparoscopy-assisted pull-through procedure for Hirschsprung’s disease using an umbilical stoma [15], and Yang et al. introduced a two-stage LAARP using an umbilical stoma for cases with a high imperforate anus [8]. In these reports, the umbilical stoma was first closed, and the navel was used as the first port. Our department performs surgeries using techniques such as temporarily closing the umbilical stoma and using the navel as the first port or inserting the first port next to the umbilical stoma. We believe that these approaches can be applied to surgeries utilizing the navel, and are currently exploring surgical techniques based on the concept of natural orifice transluminal endoscopic surgery [335].

## Conclusions

Umbilical stomas are easy to manage and result in an inconspicuous closure scar that blends into the navel’s indentation and wrinkles, leaving no surgical scars related to the stoma on the abdomen. This study yielded quantitatively excellent cosmetic results. Although patient selection is important, pediatric umbilical stomas are a good treatment option that can be actively employed.

## Data Availability

The data that support the findings of this study are available from the corresponding author, DI, upon reasonable request.
